# Macroscopic and Microscopic Properties of Some Surfactants and Biosurfactants

**DOI:** 10.3390/ijms19071934

**Published:** 2018-07-01

**Authors:** Anna Zdziennicka, Joanna Krawczyk, Katarzyna Szymczyk, Bronisław Jańczuk

**Affiliations:** Department of Interfacial Phenomena, Faculty of Chemistry, Maria Curie-Skłodowska University, Maria Curie-Skłodowska Sq. 3, 20-031 Lublin, Poland; aniaz@hektor.umcs.lublin.pl (A.Z.); j.krawczyk@poczta.umcs.lublin.pl (J.K.); katarzyna.szymczyk@poczta.umcs.lublin.pl (K.S.)

**Keywords:** surfactants, adsorption, micellization, polymers and quartz wettability

## Abstract

The adsorption of surfactants at the water-air and solid-water interfaces and their wetting properties decide their practical applications. Therefore the adsorption of monorhamnolipid, surfactin, *n*-octyl-β-d-glucopyranoside, *n*-dodecyl-β-d-glucopyranoside, *n*-dodecyl-β-d-maltoside, sucrose monodecanoate, sucrose monododecanoate, Tween 20, Tween 60, and Tween 80 at the water-air, polytetrafluoroethylene-water, polyethylene-water, poly(methyl methacrylate)-water, polyamide-water, and quartz-water interfaces, their tendency to form micelles as well as their wetting properties, were considered in the light of their microscopic properties. For this purpose, the components and parameters of the surfactant tail and head, water and solids surface tension, and surfactant contactable area with adherent medium were applied for prediction of surfactant-surfactant and surfactant-solid interactions through the water phase with regard to their adsorption, micellization, and wetting processes. Next, the Gibbs free energy of interactions was compared to the Gibbs free energy of surfactant adsorption at the water-air and solid-water interfaces as well as the micellization. It appeared that from the surfactant-surfactant and surfactant-solid interactions through the water phase determined on the basis of the tail and head of surfactant surface tension, it is possible to predict the surfactant tendency to adsorb at the water-air and solid-water interfaces, as well as to form micelles.

## 1. Introduction

In numerous surfactants applications, their adsorption at the water-air and solid-water interfaces as well as micellization play a very important role in many branches of industries and in everyday life [1,2,3,4,5,6,7,8,9,10,11,12,13,14,15,16,17,18,19,20,21,22,23,24,25,26,27]. The adsorption and micellization of surfactants occurs due to the asymmetric structure of their molecules. Thus, the surfactant molecule can be divided into two parts: hydrophobic (tail) and hydrophilic (head). The type of molecule structures that cause surfactant surface tension depends on the orientation of the molecule toward the air phase [28]. If they are oriented towards air by the tail, then the surface tension results only from the Lifshitz-van der Waals intermolecular interactions and its value should be close to that of a hydrocarbon being a tail of the surfactant. In the case when the surfactant molecules are oriented towards air phase by the head, the surface tension results from the Lifshitz-van der Waals and Lewis acid-base intermolecular interactions. The values of the surface tension of the tail and head (macroscopic property) as well as the size of the particular parts of the molecule (microscopic property) decide the surfactant tendency to adsorb at the water-air and solid-water interfaces and to form micelles at the concentration called the critical micelle concentration (CMC). During the adsorption of surfactant at the water-air interface, the tail is transferred from water to the apolar phase and can be oriented parallel or not parallel to the interface [29]. During the surfactant molecule transfer from the bulk phase to the water-air interface, the water-tail interface tension changes to the tail surface tension but the water-head interface tension can change insignificantly or be constant [30]. In the case of the adsorption of the surfactants at the solid-water interface, both parts of the surfactant molecules are practically in the aqueous phase [28]. The adsorption at this interface takes place by interactions of the tail with the solid surface through the water phase (so called hydrophobic interactions) and those of the head through the water phase. Thus, the interactions of surfactants with the solid surface should depend on the water-tail, water-head, solid-water, solid-tail, and solid-head interface tensions. In turn, the micellization process of the surfactants is connected with the transfer of the tail from the monomer to the oil phase, yet the head is in the aqueous phase before and after micellization; only its concentration is changed. Thus, the micellization process should be connected with the interactions of the tail and head of the surfactants through the water phase [28,31]. In our earlier studies it was proved that knowing the surface tension of the tail and head of straight chain surfactants, such as the homologous series of alkyl sulfate, sulphonate, chloride amines, alkyl trimethylammonium compounds and some others, it is possible to explain the constant in the Klevens equation on the basis of the interactions of these surfactants through the water phase, knowing the components and parameters of their head and tail surface tension [32,33]. It was also shown that the tendency to adsorb such kind of surfactants can be predicted on the basis of the tail-water and the tail-air interface tension [30]. As a matter of fact, this was proved not only on the basis of these tensions but also from the contactable area of the tail and head of surfactant. Such kind of considerations have not been undertaken in the case of surfactants having a greater extensive head. In turn, the relationship between the adsorption of the surfactants and the water-air interface, as well as the interactions of their molecules through the water phase to the solid surface, are difficult to find in the literature. Therefore the purpose of our study was to consider the interactions of the monorhamnolipid (RL), surfactin (SF), *n*-octyl-β-d-glucopyranoside (OGP), *n*-dodecyl-β- d-glucopyranoside (DDGP), *n*-dodecyl-β-d-maltoside (DM), sucrose monodecanoate (SMD), sucrose monododecanoate (SML), Tween 20 (T20), Tween 60 (T60), and Tween 80 (T80) molecules through the water phase as well as their interactions with polymers (polytetrafluoroethylene (PTFE), polyethylene (PE), poly(methyl methacrylate) (PMMA), polyamide (nylon 6)) and quartz through the water phase in light of the Gibbs free energy of adsorption and micellization. The studies of these interactions are based on the results obtained by us at temperature (T) equal to 293 K and published earlier [30,31,32,33,34,35,36,37,38,39,40,41,42,43,44,45,46,47,48,49,50,51,52,53,54,55].

## 2. Theory

### 2.1. Surfactants Adsorption at the Water-Air Interface

During the transfer of the surfactant molecules from the bulk phase to the surface region at the constant pressure and temperature the Gibbs free energy of the aqueous solution of the surfactants is changed [31,35,56]. This change should be connected with the surface tension of the tail ($\gamma_{T}$) and the interface tension of the water-tail ($\gamma_{WT}$), water-head ($\gamma_{WH}$) in the bulk solution as well as in the surface region ($\gamma_{WH1}$). As the Gibbs free energy ($\Delta G_{ads}$) changes due to the surfactants adsorption depend on the initial and final states, transfer of one surfactants mole from the bulk phase to air can be expressed by the equation [31,35]:

(1) $$\Delta G_{ads}=\left(\gamma_{T}-\gamma_{WT}\right)S_{T}N+\left(\gamma_{WH1}-\gamma_{WH}\right)S_{H}N$$

where $S_{T}$ and $S_{H}$ are the total contactable areas of tail and head of the surfactant molecule and *N* is the Avogadro number. If the head is not dehydrated during the transfer from the bulk phase to the surface region, Equation (1) assumes the simple form [31,35]:

(2) $$\Delta G_{ads}=\left(\gamma_{T}-\gamma_{WT}\right)S_{T}N$$

At the first approximation the $\gamma_{T}$ value is equal to the surface tension of the hydrocarbon being the hydrophobic part of surfactants molecule (tail) and $\gamma_{WT}$ one is equal to this hydrocarbon-water interface tension.

### 2.2. Surfactants Adsorption at the Solid-Water Interface

In contrast to the water-air interface, surface active molecules adsorbed at the solid-water interface remain only in the aqueous phase. This case of the surfactant adsorption at the solid-water interface occurs as a result of interactions of the tail and head of the surfactants molecules through the water phase. The Gibbs free energy of interactions ($\Delta G_{\mathrm{int}}$) of the surfactants with the solid surface through the water phase is connected with the work of adhesion of the surfactant tail ($W_{a}^{T}$) and head ($W_{a}^{H}$) to the solid surface in the aqueous solution as well as the electrostatic interactions ($\Delta G_{\mathrm{int}}^{EL}$). Thus [28,30,32,33]:

(3) $$\Delta G_{\mathrm{int}}=-\left(W_{a}^{T}+W_{a}^{H}\right)$$

$W_{a}^{T}$ can be expressed by the following equation:

(4) $$W_{a}^{T}=\gamma_{WT}+\gamma_{WS}-\gamma_{ST}$$

where $\gamma_{WS}$ and $\gamma_{ST}$ are the water-solid and solid-tail interface tensions, respectively.

However, $W_{a}^{H}$ can be expressed by the equation:

(5) $$W_{a}^{H}=\gamma_{WH}+\gamma_{WS}-\gamma_{SH}$$

As follows from Equations (3)–(5) for the system in which the electrostatic interactions between the head of surfactant molecule and the solid surface can be neglected:

(6) $$\Delta G_{\mathrm{int}}=-\left(\gamma_{WT}+\gamma_{WS}-\gamma_{ST}+\gamma_{WH}+\gamma_{WS}-\gamma_{SH}\right)$$

For one mole ($\bar{\Delta G_{\mathrm{int}}}$) there is obtained the following expression:

(7) $$\bar{\Delta G_{\mathrm{int}}}=-N\left[\left(\gamma_{WT}+\gamma_{WS}-\gamma_{ST}\right)S_{T}^{1}+\left(\gamma_{WH}+\gamma_{WS}-\gamma_{SH}\right)S_{H}^{1}\right]$$

where $S_{T}^{1}$ is the contactable area of tail at parallel orientation towards the interface plane and $S_{H}^{1}$ is the contactable area of head.

According to the van Oss et al. concept [57,58,59,60] the values of $\gamma_{WT}$, $\gamma_{WS}$, $\gamma_{ST}$, $\gamma_{WH}$ and $\gamma_{SH}$ can be calculated from the following equations:

(8) $$\gamma_{WT}=\gamma_{W}+\gamma_{T}-2\sqrt{\gamma_{W}^{LW}\gamma_{T}^{}}$$

(9) $$\gamma_{ST}=\gamma_{S}+\gamma_{T}-2\sqrt{\gamma_{S}^{LW}\gamma_{T}^{}}$$

(10) $$\gamma_{WH}=\gamma_{W}+\gamma_{H}-2\sqrt{\gamma_{W}^{LW}\gamma_{H}^{LW}}-2\sqrt{\gamma_{W}^{+}\gamma_{H}^{-}}-2\sqrt{\gamma_{W}^{-}\gamma_{H}^{+}}$$

(11) $$\gamma_{WS}=\gamma_{W}+\gamma_{S}-2\sqrt{\gamma_{W}^{LW}\gamma_{S}^{LW}}-2\sqrt{\gamma_{W}^{+}\gamma_{S}^{-}}-2\sqrt{\gamma_{W}^{-}\gamma_{S}^{+}}$$

(12) $$\gamma_{SH}=\gamma_{S}+\gamma_{H}-2\sqrt{\gamma_{S}^{LW}\gamma_{H}^{LW}}-2\sqrt{\gamma_{S}^{+}\gamma_{H}^{-}}-2\sqrt{\gamma_{S}^{-}\gamma_{H}^{+}}$$

where $\gamma_{W}^{LW}$, $\gamma_{H}^{LW}$ and $\gamma_{S}^{LW}$ are the Lifshitz-van der Waals components of the water ($\gamma_{W}$), surfactant head ($\gamma_{H}$) and solid ($\gamma_{S}$) surface tension, the indices + and − refer to the electron-acceptor and electron-donor parameters of the Lewis acid-base component ($\gamma_{}^{AB}$) of the surface tension.

### 2.3. Micellization of the Surfactants

The micellization process of the surfactants is directly connected with the Gibbs free energy of interactions of the surfactant molecules through the water phase.

The Gibbs free energy of interactions between the surfactant molecules through the water phase can be expressed in the form [28,30,32,33]:

(13) $$\Delta G_{\mathrm{int}}=\Delta G_{1W1}^{LW}+\Delta G_{1W1}^{AB}+\Delta G_{1W1}^{EL}$$

where $\Delta G_{1W1}^{LW}$ is the Gibbs free energy of interactions resulting from the Lifshitz-van der Waals forces, $\Delta G_{1W1}^{AB}$ is the Gibbs free energy of interactions resulting from the Lewis acid-base forces and $\Delta G_{1W1}^{EL}$ is the Gibbs free energy of interactions resulting from the electrostatic forces. Subscripts 1 and *W* refer to the surface active ions of surfactant and water, respectively. 

If we treat the interactions of tail and head of the surfactant molecules separately, Equation (14) can be written:

(14) $$\Delta G_{\mathrm{int}}=\Delta G_{TWT}^{LW}+\Delta G_{TWT}^{AB}+\Delta G_{HWH}^{LW}+\Delta G_{HWH}^{AB}+\Delta G_{HWH}^{EL}$$

Because

(15) $$\Delta G_{TWT}^{LW}+\Delta G_{TWT}^{AB}=-2\gamma_{TW},\;\mathrm{and}\;\Delta G_{HWH}^{AB}+\Delta G_{HWH}^{EL}=-2\gamma_{HW}$$

we obtain:

(16) $$\Delta G_{\mathrm{int}}=-2\left(\gamma_{TW}+\gamma_{HW}\right)+\Delta G_{HWH}^{EL}$$

The Gibbs free energy of interactions dealing with one mole of surfactant ($\bar{\Delta G_{\mathrm{int}}}$) fulfils the equation:

(17) $$\bar{\Delta G_{\mathrm{int}}}=-2\left(\gamma_{TW}S_{T}^{1}N+\gamma_{HW}S_{H}^{1}N\right)+\Delta G_{HWH}^{EL}S_{H}^{1}N$$

If there are no electrostatic interactions or they are very weak, Equation (17) has the form [30,32,33]:

(18) $$\bar{\Delta G_{\mathrm{int}}}=-2\left(\gamma_{TW}S_{T}^{1}N+\gamma_{HW}S_{H}^{1}N\right)$$

### 2.4. Calculations of Volume and Contactable Area of Surfactants Molecule

To determine the molecule volume the length of the bonds between different atoms and the angle between them as well as the average distance between the molecules at a given temperature must be known. In our earlier papers [32,33,38,46] we suggest that the volume of a given surfactant can be expressed by the sum of cubes in which the particular parts of the surfactant are inscribed. Thus, for calculations of the cube volume the knowledge of the cube size is necessary. For example, if the hydrophobic part of surfactant is a straight chain of alkane, we propose that its volume at *T* = 293 K can be calculated from the following expression:

(19) $$V_{T}=4.6^{2}L_{T}$$

where 4.6 = 2.6 + 2 (2.6 is the width of alkyl chain and 2 is the average distance between the chains at *T* = 293 K).

$L_{T}$ fulfils the equation [31]:

(20) $$L_{T}=0.92+2+1.27(n-1)$$

where *n* is the number of carbon atoms of the chain.

On the other hand, for calculations of the volume of straight alkyl chain (tail), Tanford proposed the following expression [61]:

(21) $$V_{T}=27.4+26.9n$$

However, the length of alkyl chain ($L_{T}$) should be established from the expression:

(22) $$L_{T}=1.5+1.265n$$

The usefulness of Tanford and our methods [31,61] can be examined by the calculation of alkanes molecule volume and a comparison to those obtained from the molar weight of the alkanes and their density.

It results from the Tanford method [61] that the volume of the alkane molecule ($V_{A}$) gives the following equation:

(23) $$V_{A}=54.8+26.9n$$

The alkane volume can be also obtained from our equation, which has the form [31]:

(24) $$V_{A}=4.6^{2}\left[1.84+2+1.27(n-1)\right]$$

The good agreement between the molecule volume of 11 alkanes calculated from Equations (23) and (24) and those obtained from alkanes density (Table 1) indicates that our method can be applied for determination of the tail of surfactants volume and their contactable area. Knowing the size of the cube describing the volume of the hydrophobic part of the surfactant molecule, it is very easy to determine its contactable area.

Our studies also indicate that the hydrophilic part of the surfactants molecule volume ($V_{H}$) and contactable area ($S_{H}$) can be determined in the same way as the hydrophobic part of this molecule. However, it should be mentioned that for our calculations the average distance between the hydrophilic part of the surfactant molecule and water one was assumed to be equal to the length of the hydrogen bonds (1.93 Å). Obviously, in the case of surfactants molecules composed of different groups, the calculation of molecule volume on the basis of the cubes is more complicated.

## 3. Calculations and Discussion

### 3.1. Packing of the Surfactants in the Surface Layers at the Water-Air Interface

The maximal packing of the surfactants molecules in the monolayer depends on their contactable area. In the case of the perpendicular orientation towards the interface of the surfactant molecules, the contactable area in the monolayer is equal to the cross section area of the hydrophilic part of the molecules. It is the so called limiting area [29,56,62]. However, at the parallel orientation of the surfactant molecules in the surface monolayer, the limiting area is equal to the contactable area at this orientation, which depends on the size of the hydrophobic and hydrophilic parts of the surfactant molecules. At the perpendicular orientation of surfactant molecules, this area is difficult to obtain in practice because of the repulsive forces between the hydrophilic parts of the surfactant molecules. Thus, the maximal packing of the surfactant molecules in the monolayer depends on the difference between the attractive and repulsive forces between them. The packing of the surfactant molecules can be expressed by the ratio of the Gibbs surface excess concentration of the surfactant to the limiting concentration in the surface layer. Thus:

(25) $$X_{1}^{S}=\frac{\Gamma_{1}}{\Gamma_{1}^{\infty }}$$

where $X_{1}^{S}$ is the fraction of surface occupied by the surfactant molecules, $\Gamma_{1}$ is the Gibbs surface excess concentration of surfactant in the monolayer and $\Gamma_{1}^{\infty }$ is the limiting surface concentration. 

Obviously, it was assumed that $\Gamma_{1}$ is equal to the total number of the surfactant moles occupying the unity surface area in the monolayer. The $\Gamma_{1}^{\infty }$ value depends on the orientation of surfactant molecules in the surface monolayers. The part of the surface occupied by the water molecules can be expressed:

(26) $$X_{o}^{S}=\frac{\Gamma_{o}}{\Gamma_{o}^{\infty }}$$

where $X_{o}^{S}$ is the fraction of the surface occupied by water molecules, $\Gamma_{o}$ is the number of the water molecules in the unity surface area and $\Gamma_{o}^{\infty }$ is the maximal number of water molecules in the unity surface area. Because:

(27) $$X_{1}^{S}+X_{o}^{S}=1$$

thus:

(28) $$\Gamma_{o}=\frac{\Gamma_{1}^{\infty }-\Gamma_{1}}{K}$$

where $K=\frac{\Gamma_{1}^{\infty }}{\Gamma_{o}^{\infty }}$.

Knowing $\Gamma_{o}$ $X_{1}^{S}$ can be expressed in a different way [46]:

(29) $$X_{1}^{S}=\frac{\frac{\Gamma_{1}}{\Gamma_{1}^{\infty }}}{\frac{\Gamma_{1}}{\Gamma_{1}^{\infty }}+\frac{\Gamma_{o}}{\Gamma_{o}^{\infty }}}=\frac{\Gamma_{1}}{\Gamma_{1}+K\Gamma_{o}}$$

In the case $\Gamma_{1}^{\infty }=\Gamma_{0}^{\infty }$:

(30) $$X_{1}^{S}=\frac{\Gamma_{1}}{\Gamma_{1}+\Gamma_{o}}$$

Equation (30), very often applied for the determination of the surface area fraction occupied by surfactant molecules, is generally not fulfilled.

To calculate the surface area fraction occupied by the surfactant molecules in the monolayer the limiting concentration of the surfactants in the saturated monolayer is needed. Among others, the limiting concentration can be determined from the Joos equation, which has the form [62]:

(31) $$\exp\left(\frac{-\Pi }{RT\Gamma_{o}^{\infty }}\right)+\exp\left(\frac{-\Pi }{nRT\Gamma_{1}^{\infty }}\right)\frac{C}{a_{1}}=1$$

where $\Pi =\gamma_{W}-\gamma_{LV}$, $\gamma_{W}$ is the water surface tension, $\gamma_{LV}$ is the surfactant solution surface tension, $a_{1}=\exp\left(\frac{\mu_{S}^{0S}-\mu_{S}^{0B}}{RT}\right)\omega$ ($\mu_{S}^{0S}$ and $\mu_{S}^{0B}$ are the standard potentials of surfactant in the surface layer and bulk phase), *ω* is the number of water moles in 1 dm^3^ and *n* = 1 for the nonionic surfactant and *n* = 2 for the ionic surfactant 1:1 AB electrolyte type.

The values of $\Gamma_{1}^{\infty }$ for RL, SF, OGP, DDGP, DM, SMD, SML, T20, T60, and T80 calculated from Equation (31) are presented in Table 2 [39,46,50,51,54]. The $\Gamma_{1}^{\infty }$ values for these surfactants were also determined on the basis of the cross section area of the hydrophilic part of the surfactant molecule, which was found from the basis of the bonds length and the angle between them as well as the average distance between the molecules.

Taking into account the maximal Gibbs surface excess concentration of the surfactants at the water-air interface and the limiting concentration of the surfactants in the monolayer determined in both ways the maximal $X_{1}^{S}$ values were calculated from Equation (25) (Table 3). As follows from Table 3 for the studied surfactants the fraction of surface area occupied by the surfactant molecule calculated on the basis of $\Gamma_{1}^{\max}$ and $\Gamma_{1}^{\infty }$ obtained from the Joos equation [62] is in the range from 0.7686 to 0.9752. These values are considerably higher than those determined from Equation (30) for the classical anionic, cationic, and nonionic surfactants (Table 3). This means that there are no weak repulsive interactions, even for the ionic biosurfactants (RL and SF), which probably results from hydrogen bonds formation between the hydrophilic parts of the surfactant molecules. It should be also stressed that the ratio of the surfactant area ($X_{1}^{S}$) for the surfactant having the same head depends on the contactable area of the surfactant tail and that in the case of SF, T20, T60, and T80 the ratio of surface area occupied by their molecules probably depends on the configuration of the hydrophilic part of their molecules. For the same reason, it is not possible to obtain some values of $\Gamma_{1,theor}^{\infty }$ and $A_{o,theor}^{}$ except for their range. In the case of disaccharide-based surfactants, the $X_{1}^{S}$ values probably depend on the mutual position of the sugar groups in the surface region. 

It can be expected that on the basis of the surface area fraction occupied by the surfactant and water, it is possible to predict the minimal surface tension value for the aqueous solution of the surfactants. If we assume that in the saturated monolayer at the water-air interface the surfactant molecules are oriented perpendicularly towards the interface and directed to the air phase with the tail, at the first approximation, the surface tension can be expressed by the following equation:

(32) $$\gamma_{LV}=X_{1}^{S}\gamma_{T}+X_{o}^{S}\gamma_{W}$$

The values of $\gamma_{LV}$ for the aqueous solutions of all studied surfactants calculated from Equation (32) are consistent with those measured after CMC (Table 4) [38,39,46,50,51,54]. Thus knowing the maximal packing of the surfactant molecules it is possible to predict the maximal reduction of the water surface tension by the adsorbed molecules of the surfactant at the water-air interface. This means that the effectiveness of the surfactants adsorption is strictly connected with the maximal reduction of the water surface tension by the given surfactant. However, it should be stressed that the minor agreement between the theoretically calculated and measured values of minimal surfactant solutions surface tension was obtained in the case of Tween’s surfactants, which may result from a different conformation of their molecules at the interface. In such a case, it is difficult to determine precisely the values of the fraction of surface area occupied by the surfactant molecules.

### 3.2. Packing of Surfactants in the Surface Layers at the Solid-Water Interface

The mechanism of adsorption, packing and orientation of the surfactant molecules in the formed layer at the solid-water interface is more complicated than those of the water-air one. In the case of the solid-water system, the molecules adsorbed at the interface do not transfer to the solid phase and must remove water molecules from the solid surface. As a result of adsorption, the monolayer or bilayer can be formed at the solid-water interface, depending on the kind of surfactant and solid. According to van Oss et al. [57,58,59,60], solids can be divided into apolar, monopolar, and bipolar. The surface tension of apolar and monopolar solids results practically from the Lifshitz-van der Waals intermolecular interactions; however, in the case of the monopolar ones the Lewis acid-base interactions between their surface and surfactant molecules can take place [43,44,45]. In the case of bipolar solids, their surface tension results from the Lifshitz-van der Waals and Lewis acid-base interactions. Knowing the amount of surfactant adsorbed at the solid-water interface and the contactable area of the surfactant tail and head it is possible to determine the fraction of the area occupied by the surfactant molecules as well as to establish their orientation and packing in the surface layer. As a matter of fact, it is impossible to determine directly the surfactant concentration in the surface region on the flat solid surface. In such case the Gibbs surface excess concentration can be determined from the isotherm of the contact angle of the aqueous solution on the solid surface [48,49,55]. Our studies show that the adsorption of RL, SF, OGP, DDGP, DM, SMD, SML, T20, T60, and T80 at the PTFE-water and PE-water interfaces is comparable to that at the water-air interface (Table 2 and Table 5) [48,49,55]. This means that the perpendicular orientation of the molecules takes place at the saturated monolayer.

This was confirmed by the maximal Gibbs surface excess concentration values and by limiting excess concentration calculated from the Joos equation [62], as well as by the mole fraction of the area occupied by the surfactant molecules in the saturated monolayer, which are close to those at the water-air interface (Table 2). Thus, in the saturated monolayer the molecules of these surfactants are oriented perpendicularly to the polymer-water interface or at an angle somewhat different from 90°. Obviously, the minimal area occupied by the molecule of these surfactants is close to the limiting area calculated from the bonds length, the angle between them, and the average distance between the molecules [38,39,44,46,47,49]. This indicates that there are weak repulsive interactions between the molecules in the monolayer. Of the studied surfactants, RL and SF can be treated as ionic ones. However, their behavior in the surface monolayer is similar to that of nonionic ones [29,35,38,46]. This suggests that it is possible to form hydrogen bonds between the –OH and =CO groups present in the hydrophilic part of RL and SF molecules.

It should be mentioned that in the case of SF the fraction of the area occupied by its molecules in the saturated monolayer is lower than that of the RL one determined from both the values of the limiting area calculated from the Joos equation [62] and the minimal cross section area of the hydrophilic part. However, this part of the SF molecule can change its configuration, which causes the increase of its cross section area. Taking into account the maximal cross section area of the head of the SF molecules, the calculated surface area fraction is close to unity. Thus, most likely the monolayer formed by the SF at the PTFE-water and PE-water interfaces is closely packed and there are no repulsive interactions between its molecules, despite its ionic character.

The adsorption of the studied surfactants at the PMMA-water, nylon 6-water, and quartz-water interfaces is considerably lower than the hydrophobic polymer-water one. The adsorption decreases with the increasing solid polarity [48,49,55]. At the monopolar PMMA-water interface the minimal surface area occupied by RL and SF is higher than the minimal contactable area of their molecules at their parallel orientation (Table 5). From the fraction of the area occupied by these molecules on the assumption of their parallel orientation, it results in lower than unity and lower for the SF than for the RF. However, as mentioned above, the SF hydrophilic part can change its configuration. In such a case the fraction of the area covered by the SF molecules can be larger than when it results from $\Gamma_{1}^{\max}$/$\Gamma_{1,theor}^{\infty }$($\Gamma_{1,theor}^{\infty }$—calculated from the molecule size). Perhaps the water molecules hydrate the surfactant head more strongly than the PMMA surface. In such a case the monolayer is completely covered by the parallel oriented surfactant molecules hydrated by the water ones.

It should be remembered that it is difficult to predict the contactable area of these surfactants precisely on the basis of the length of the bonds, the angle between them, and the average distance between the molecules because their contactable area depends largely on their configuration and hydrophilic hydration. However, independently of the RL and SF molecules configuration the minimal surface area occupied by their molecules corresponding to the saturated monolayer is smaller than their contactable area. In the case of the bipolar solids, such as nylon 6 and quartz, the adsorption of the RL and SF is smaller than in the PMMA-solution system and their molecules are oriented parallel towards the interface, but the fraction of the surface area occupied by the RL and SF molecules at the nylon 6-water and quartz-water interfaces is smaller than in the case of the PMMA-water one. However, it should be remembered that the area could be larger than that that calculated if the water is taken into account in determination of the $\Gamma_{1}^{\max}$/$\Gamma_{1,theor}^{\infty }$ ratio (Table 6), as mentioned above.

In the case of PMMA, nylon 6, and quartz, the orientation is closer to the parallel. It is possible that the sugar surfactants having two sugar rings in the molecule can contact with one or two rings with the solids surface. As a matter of fact, this influences the contactable area of the sugar surfactants with solid surface. If all studied sugar surfactants are assumed to be in contact with PMMA, nylon 6, and quartz surface with one sugar ring than in any case the minimal area occupied by the sugar surfactants on PMMA, nylon 6, and quartz-water interfaces is close or higher than the theoretical one (Table 2). The best agreement between the surface area occupied by one surfactant molecule at the solid-water interface calculated from the bonds length and the angle between them, as well as the average distance between molecules and that calculated for the saturated monolayer, is for PMMA. In contrast to RL and SF for PMMA, the sugar surfactant formed a more densely packed monolayer, which indicated that their molecules are less hydrated than those of RL and SF. The sugar surfactants having two sugar rings in their molecule probably form the monolayer at the nylon 6 and quartz surface in which their molecules are contacted with two sugar rings because their minimal surface area occupied by one molecule in the saturated monolayer is closer to the theoretical one calculated on the assumption that the sugar rings are in contact with the solid surfaces. As for nylon 6 and quartz, the hydrophilic part of the sugar surfactants is rather oriented towards the bulk phase which is confirmed by the contact angle values, which especially for quartz are lower than for PMMA. In the case of PMMA, the orientation of that part is rather reversed.

In the case of the Tweens in the saturated monolayer at the PMMA-water interface, their molecules are contacted with the PMMA surface by a part of the surfactant molecules and the head of their molecules is directed towards the water phase. The increase of solid polarity from nylon 6 to quartz causes a decrease in the amount of Tweens adsorbed at the solid-water interface and in every case the minimal area occupied by the surfactants molecule at this interface is larger than the contactable area at the orientation parallel to the interface calculated from the bonds length, the angle between them, and the average distance between molecules.

### 3.3. Gibbs Free Energy of Adsorption and Surface Tension of Surfactant Tail and Head

Positive and negative interactions between the surfactant molecules in the saturated monolayer at the water-air interface, the size of the hydrophilic group of surfactant molecules, and the surface tension of the hydrocarbon being the tail of the surfactant molecules decide about the packing of the surfactant molecules in the monolayer and reduction of the water surface tension. However, the standard Gibbs free energy of adsorption gives information about the efficiency of adsorption of a given surfactant at the water-air interface. The literature reports many methods for the determination of the standard Gibbs free energy of adsorption ($\Delta G_{ads}^{o}$) [29,46,56]. Most of them are based on the constant value in the Langmuir isotherm equation [29,56,63]. Some of them result from the consideration of chemical potential in the monolayer and bulk phase in the equilibrium state. However, we proposed that the standard Gibbs free energy of adsorption depends on the surface tension of surfactants tail and head and the size of their molecule. To examine whether it works in the case of the studied surfactants, the $\Delta G_{ads}^{o}$ values were calculated from Equation (2). Thus, it was assumed that during the transport of the surfactant molecules from the bulk phase to the surface region the head-water interface tension is constant and the tail is oriented parallel to the water-air interface. In such case one side of surfactant molecule is in contact with the water molecules (Table 7). The $\Delta G_{ads}^{o}$ values calculated in this way are at the first approximation close to those determined by applying the Langmuir equation modified by de Boer [63]. The $\Delta G_{ads}^{o}$ values were calculated from this equation using *RT* for all ionic and nonionic surfactants. Application of *2RT* in this equation is not thermodynamically justified [46]. Applying *2RT* for ionic surfactants, the calculated $\Delta G_{ads}^{o}$ are considerably lower than those for the nonionic ones which would indicate that adsorption efficiency of the ionic surfactants is higher than that for the nonionic ones. From the comparison of $\Delta G_{ads}^{o}$ values calculated from the Langmuir equation to those based on the surface tension of tail and water-tail interface tension it results that on the basis of the surface tension of the hydrocarbon being the tail in the surfactants molecule it is possible to predict the surfactants tendency to adsorb at the water-air interface.

In the case of the surfactants adsorption at the solid-water interface, it is impossible to predict their tendency to adsorb only from the surface tension of the surfactant tail. However, this tendency should be connected with the Gibbs free energy of the interactions between the surfactants molecule and the solid surface through the water phase.

For this purpose, the energy was calculated from Equation (7). The obtained results were compared to the Gibbs standard free energy of adsorption calculated from the Langmuir [29,56,63] and our equations. The equation proposed by us has the following form [41,48]:

(33) $$\Delta G_{ads}^{o}=RT\left(\ln CMC-\ln\omega \right)-\frac{\gamma_{LV}\cos\theta_{S}-\gamma_{W}\cos\theta_{W}}{\Gamma_{1}^{\max}}$$

where: $\theta_{S}$ and $\theta_{W}$ are the contact angles of the surfactant solution at CMC and water, respectively. The values of $\Delta G_{ads}^{o}$ calculated from the Langmuir equation for each studied surfactant practically do not depend on the kind of solid (Table 8). Contrary to these values those of $\Delta G_{ads}^{o}$ calculated from the contact angle of water and solution at CMC depend on the solid type. It is interesting that the values of the Gibbs free energy of interactions between the surfactant molecules and the solid surface through the water phase are closer to the $\Delta G_{ads}^{o}$ values calculated on the basis of the contact angle of water and solution at CMC rather than those determined from the Langmuir equation. This indicates that at the first approximation it is possible to predict the surfactant tendency to adsorb on the solid surface by applying the surface tension values of the surfactants tail and head and their components and parameters.

### 3.4. Surface Tension of Surfactant Tail and Head and Their Tendency to Form Micelles

The adsorption process of the surfactants at the interface is connected with that of micellization. However, there is no direct relation between the micellization and adsorption processes. The standard Gibbs free energy of micellization ($\Delta G_{mic}^{o}$) is a measure of the surfactants tendency to form the micelles at a proper concentration. In the literature it is possible to find many methods for the determination of $\Delta G_{mic}^{o}$. In our previous paper it was proved that whether the ionic or nonionic surfactants form the micelles, $\Delta G_{mic}^{o}$ fulfils the following simple equation [29]:

(34) $$\Delta G_{mic}^{o}=RT\ln\frac{CMC}{\omega }$$

Obviously, it is assumed that the surfactants activity at the CMC is close to unity.

On the other hand, the micellization process should be connected with the surfactant molecules interactions through the water phase and their Gibbs free energy ($\Delta G_{\mathrm{int}}^{o}$) should be close to $\Delta G_{mic}^{o}$. The $\bar{\Delta G_{\mathrm{int}}}$ values were calculated from Equation (18) for RL, SF, OGP, DDGP, DM, SDM, SLM, T20, T60, and T80 [47,50,52,53]. It proved that the calculated values are close to the $\Delta G_{mic}^{o}$ ones (Table 9). For the calculations, it was assumed that the water molecules can penetrate to the third or fourth –CH_2_– groups in the micelle core. The depth of the water molecules penetration into the micelle core depends on the kind of the surfactant hydrophilic part [29]. As a matter of fact, we should remember that during the micellization process the partial molar volume of the surfactant is changed. These changes depend on the average distance between the water and surfactant molecules in relation to the distance between the surfactant molecules in the micelle. It was shown that, assuming the minimal distance equal to 1.56 Å, it is possible to predict the partial molar volume of surfactants in the water phase and for 2 Å in the micelle at 293 K. The values of partial molar volume of surfactants calculated in this way are presented in Table 10 together with those calculated from the density of aqueous solutions of surfactant. It appeared that density of aqueous solutions of studied surfactants can be described by two linear functions as the percentage concentration function before and after CMC. Two values of partial molar volumes were determined based on these functions. These values are more or less comparable to the partial molar volume of surfactant calculated from the bond length and the angle between them as well as the average distance between the tail and water molecules equal to 1.56 Å and between the tails equal to 2 Å. The best agreement between the theoretical partial molar volume of the surfactants and those obtained on the basis of the density measurements was obtained for RL and SF where the greatest changes were observed before and after CMC. In the case of the Tweens, the partial molar volume of the surfactant calculated, based on the density, is smaller than those calculated theoretically, which is probably connected with the changes in dehydration of the polyoxytethylene chains or the surfactant molecule conformation. 

## 4. Conclusions

It can be stated:

The Gibbs standard free energy of the adsorption of the surfactant at the water-air interface can be predicted on the basis of the surface tension of the surfactant tail, the water-tail interface tension, as well the surfactant tail area contactable with the water molecules. These predicted values are closer to the data obtained in the literature, applying different methods for monorhamonolipid and sucrose surfactants.

There is the relation between the surfactants molecule interactions through the water phase with the solid surface and the standard Gibbs free energy of adsorption at the solid-water interface. The best agreement between the standard Gibbs free energy of adsorption and the interactions are observed for the all studied surfactants at the PTFE/PE-water interface.

The standard Gibbs free energy of micellization can be predicted based on the surface tension of the surfactants tail and head, its components and parameters.

The partial molar volume of the surfactant in the monomeric and aggregation forms can be predicted from the size of the molecule calculated from bonds length, the angle between them and the average distance between the surfactants and the water molecules as well as between the surfactants molecules.

## Figures and Tables

**Table 1:** The molecule volume of alkanes calculated based on the bonds length, the angle between them, and the average distance between the molecules equal to 2 Å as well as from the density at *T* = 293 K.

Alkane	Molecule Volume from Equation (23) [Å^3^]	Molecule Volume from Equation (24) [Å^3^]	Molecule Volume from Density [Å^3^]
hexane	216.20	215.62	215.17
heptane	243.10	242.49	243.24
octane	270.00	269.37	269.78
nonane	296.90	296.24	296.45
decane	323.80	323.11	323.60
undecane	350.70	349.99	350.71
dodecane	377.60	376.86	377.09
tridecane	404.50	403.73	404.97
tetradecane	431.40	430.61	432.27
pentadecane	458.30	457.48	458.33
heksadecane	485.20	484.35	485.13

**Table 2:** The values of maximal ($\Gamma_{1}^{\max}$), limiting ($\Gamma_{1}^{\infty }$) and theoretical limiting ($\Gamma_{1,theor}^{\infty }$) Gibbs surface excess concentration of the surfactant at the water-air interface as well as the minimal (A), limiting ($A_{0}$) and theoretical limiting ($A_{0,theor}$) area occupied by one surfactant molecule at *T* = 293 K, with the exception of DDGP (*T* = 298 K).

Surfactant	$\Gamma_{1}^{\max}$ [·10^–6^ mol/m^2^]	$A_{}$ [Å^2^]	$\Gamma_{1}^{\infty }$ [·10^–6^ mol/m^2^]	$A_{0}$ [Å^2^]	$\Gamma_{1,theor}^{\infty }$ [·10^–6^ mol/m^2^]	$A_{0,theor}$ [Å^2^]
RL	2.01	82.60	2.403	69.09	2.403	69.09
SF	1.38	120.31	1.782	93.17	1.38–1.78	93.17–120.24
OGP	3.64	45.61	4.34	38.26	4.74	35.05
DDGP	4.34	38.26	4.50	36.90	4.74	35.05
DM	3.28	50.62	3.78	43.92	4.74	35.05
SMD	3.18	52.21	3.50	47.50	4.74	35.05
SML	3.10	53.56	3.42	48.50	4.74	35.05
T20	2.79	59.51	3.63	45.74	2.45–4.84	34.30–67.64
T60	3.00	55.34	3.61	45.99	2.45–4.84	34.30–67.64
T80	3.94	42.14	4.04	41.10	2.45–4.84	34.30–67.64

RL and SF [38,46]; OGP, DDGP, DM, SMD and SML [39,51,54]; T20, T60, and T80 [50].

**Table 3:** Maximal ($\Gamma_{1}^{\max}$) and limiting ($\Gamma_{1}^{\infty }$) surfactant Gibbs surface excess concentrations, minimal (A) and excluded areas ($A_{o}$) [35] as well as the fraction of surface area occupied by the surfactant molecule ($X_{1}^{S}$) at the water-air interface calculated from $\Gamma_{1}^{\max}$ /$\Gamma_{1}^{\infty }$ for the classical surfactants at the water-air interface at *T* = 293 K.

Surfactant	$\Gamma_{1}^{\max}$ [·10^–6^ mol/m^2^]	$A_{}$ [Å^2^]	$\Gamma_{1}^{\infty }$ [·10^–6^ mol/m^2^]	$A_{o}$ [Å^2^]	$X_{1}^{S}$
TX-100	2.83	58.67	4.65	35.70	0.6085
TX-114	2.52	65.89	4.65	35.70	0.5419
TX-165	2.12	78.32	4.65	35.70	0.4558
SDDS	3.20	51.88	4.74	35.00	0.6746
SHS	2.96	56.09	5.93	28.00	0.4992
SDSa	2.30	72.19	4.78	34.77	0.4817
CTAB	3.10	53.56	5.45	30.46	0.5687
CPyB	2.60	63.86	4.27	38.88	0.6089
DDEAB	2.60	63.86	6.13	27.08	0.4241
TTAB	3.20	51.88	5.43	30.58	0.5894
BDDAB	1.60	103.77	4.33	38.30	0.3691

**Table 4:** The values of the surface area fraction occupied by surfactants molecule in the saturated monolayer ($X_{1}^{S}$), minimal surface tension of aqueous solution of surfactants as well as tail surface tension ($\gamma_{T}$) at *T* = 293 K.

Surfactant	$X_{1}^{S}$		$\gamma_{T}$ [mN/m]	$\gamma_{LV}$ Minimal Measured [mN/m]	$\gamma_{LV}$ Minimal Theoretical [mN/m] c	$\gamma_{LV}$ Minimal Theoretical [mN/m] d
	a	b				
RL	0.836454	0.836454	21.80	27.89	30.14	30.14
SF	0.774411	1.000000	24.70	32.37	35.55	35.55
OGP	0.882629	0.793249	21.80	29.84	27.79	32.34
DDGP	0.964444	0.915612	25.08	28.50	26.78	29.11
DM	0.867725	0.691983	25.08	35.25	31.39	39.78
SMD	0.908571	0.670886	22.91	35.50	27.47	39.33
SML	0.870787	0.654008	24.70	37.22	30.92	41.34
T20	0.768595	0.576446	24.70	34.90	35.83	45.07
T60	0.831025	0.619835	26.90	37.85	34.66	44.35
T80	0.975248	0.814050	26.90	39.50	28.04	35.44

a: Calculated values of $X_{1}^{S}$ using $\Gamma_{1}^{\infty }$ determined from the Joos equation; b: Calculated values of $X_{1}^{S}$ using $X_{1}^{S}$ obtained from the length of bonds and the angle between them as well as the average distance between molecules in water; c: Calculated on the basis of $X_{1}^{S}$ (data a); d: Calculated on the basis of $X_{1}^{S}$ (data b).

**Table 5:** The values of maximal ($\Gamma_{1}^{\max}$), limiting ($\Gamma_{1}^{\infty }$) and theoretical limiting ($\Gamma_{1,theor}^{\infty }$) Gibbs surface excess concentrations of surfactant at the solid-water interface as well as the minimal ($A_{}$), limiting ($A_{0}$) and theoretical limiting ($A_{o,theor}^{}$) areas occupied by one surfactant molecule at *T* = 293 K, with the exception of DDGP (*T* = 298 K).

Surfactant	Solid	$\Gamma_{1}^{\max}$ [·10^–6^ mol/m^2^]	A [Å^2^]	$\Gamma_{1}^{\infty }$ [·10^–6^ mol/m^2^]	$A_{0}$ [Å^2^]	$\Gamma_{1,theor}^{\infty }$ [·10^–6^ mol/m^2^]	$A_{o,theor}^{}$ [Å^2^]
RL	PTFE	1.98	82.60	2.28	72.82	2.403	69.09
	PE	2.01	82.60	2.12	78.32		
	PMMA	0.71	233.85	0.91	182.45	1.04	159.38
	nylon 6	0.60	276.72	0.81	204.98		
	Quartz	0.34	488.32	0.47	353.26		
SF	PTFE	1.34	123.90	1.75	94.87	1.38–1.782	93.14–120.24
	PE	1.34	123.90	1.75	94.87		
	PMMA	0.55	301.87	1.10	150.94	1.06	163.52
	nylon 6	0.43	386.12	0.9	184.48		
	Quartz	0.45	368.96	0.87	190.84		
OGP	PTFE	3.82	43.46	4.34	38.26	4.74	35.05
	PE	1.84	90.23	4.34	38.26		
	PMMA	1.80	92.24	2.18	76.12	1.96	84.78
	nylon 6	1.50	110.69	1.96	84.70		
	Quartz	1.04	159.65	1.99	83.50		
DDGP	PTFE	4.27	38.88	4.52	36.74	4.74	35.05
	PE	3.41	48.69	4.59	36.19		
	PMMA	1.53	108.52	1.98	83.85	1.54	107.87
	nylon 6	1.40	118.65	1.52	109.00		
	Quartz	0.90	184.48	1.46	113.98		
DM	PTFE	3.30	50.31	4.56	36.42	4.74	35.05
	PE	3.21	51.72	4.61	36.00		
	PMMA	1.51	109.95	2.71	61.30	1.54/1.24	107.87/133.75
	nylon 6	1.16	143.13	1.52	109.00		
	Quartz	0.91	182.45	1.60	103.89		
SMD	PTFE	3.12	53.21	4.65	35.67	4.74	35.05
	PE	3.08	53.91	4.49	37.00		
	PMMA	1.53	108.52	2.26	73.50	1.55/1.25	107.00/132.87
	nylon 6	1.25	132.82	1.58	105.33		
	Quartz	0.85	195.33	1.55	107.00		
SML	PTFE	3.11	53.39	4.50	36.86	4.74	35.05
	PE	3.03	54.80	4.50	36.86		
	PMMA	1.47	112.95	2.75	60.31	1.54/1.24	108.14/134.02
	nylon 6	1.21	137.22	1.52	109.51		
	Quartz	0.81	204.98	1.55	107.28		
T20	PTFE	2.87	57.85	3.73	44.50	2.45–4.84	34.30–67.64
	PE	2.94	56.40	3.83	43.40		
	PMMA	1.92	86.47	2.53	65.70	0.99	167.50
	nylon 6	1.29	129.11	1.74	95.20		
	Quartz	0.69	241.67	0.97	172.05		
T60	PTFE	2.94	56.45	3.73	44.57	2.45–4.84	34.30–67.64
	PE	2.97	55.88	3.69	45.03		
	PMMA	2.08	79.82	2.69	61.81	0.70	236.20
	nylon 6	1.454	114.19	1.93	85.98		
	Quartz	0.86	193.96	1.17	141.66		
T80	PTFE	3.41	48.65	4.14	40.10	2.45–4.84	34.30–67.64
	PE	3.35	49.58	4.06	40.89		
	PMMA	2.40	69.21	3.48	47.76	0.70	236.20
	nylon 6	1.67	99.42	2.51	66.17		
	Quartz	1.03	161.04	1.60	103.83		

RL and SF [40,41,48]; OGP, DDGP, DM, SMD and SML [40,41,55]; T20, T60 and T80 [49].

**Table 6:** The values of the surface area occupied by surfactant molecules at the solid-water interface at *T* = 293 K.

Surfactant	Solid	$\Gamma_{1}^{\max}$ $/\Gamma_{1}^{\infty }$	$\Gamma_{1}^{\max}$ $/\Gamma_{1,theor}^{\infty }$
RL	PTFE	0.8684	0.8240
	PE	0.9500	0.8365
	PMMA	0.7802	0.6827
	nylon 6	0.7407	0.5769
	Quartz	0.7234	0.3269
SF	PTFE	0.7657	0.9710–0.7520
	PE	0.7657	0.9710–0.7520
	PMMA	0.5000	0.5189
	nylon 6	0.4778	0.4057
	Quartz	0.5172	0.4245
OGP	PTFE	0.8802	0.8059
	PE	0.424	0.3882
	PMMA	0.8257	0.9184
	nylon 6	0.7653	0.7653
	Quartz	0.5226	0.5306
DDGP	PTFE	0.9447	0.9008
	PE	0.7429	0.7194
	PMMA	0.7727	0.9935
	nylon 6	0.9211	0.9091
	Quartz	0.6164	0.5844
DM	PTFE	0.7237	0.6962
	PE	0.6963	0.6772
	PMMA	0.5572	1.2177
	nylon 6	0.7632	0.9355
	Quartz	0.5688	0.7339
SMD	PTFE	0.6710	0.6582
	PE	0.6860	0.6498
	PMMA	0.6770	1.2240
	nylon 6	0.7911	1.0000
	Quartz	0.5484	0.6800
SML	PTFE	0.6911	0.6561
	PE	0.6733	0.6392
	PMMA	0.5345	1.1855
	nylon 6	0.7961	0.9758
	Quartz	0.5226	0.6532
T20	PTFE	0.7694	1.1714–0.5930
	PE	0.7676	1.2000–0.6074
	PMMA	0.7589	1.9394
	nylon 6	0.7414	1.3030
	Quartz	0.7113	0.6970
T60	PTFE	0.7882	1.2000–0.6074
	PE	0.8049	1.2122–0.6136
	PMMA	0.7732	2.9714
	nylon 6	0.7534	2.0771
	Quartz	0.7350	1.2286
T80	PTFE	0.8237	1.3918–0.7045
	PE	0.8251	1.3673–0.6921
	PMMA	0.6897	3.4286
	nylon 6	0.6653	2.3857
	Quartz	0.6438	1.4714

**Table 7:** The values of the standard Gibbs free energy of adsorption of surfactants ($\Delta G_{ads}^{o}$) at the water-air interface taken from the literature and calculated from Equation (2) at *T* = 293 K.

Surfactant	$\Delta G_{ads}^{o}$[kJ/mol] Literature Data	$\Delta G_{ads}^{o}$[kJ/mol] Equation (2)
RL	−42.52	−42.96
SF	−47.29	−42.45
OGP	−28.64	−30.06
DDGP	−42.46	−40.43
DM	−39.22	−40.43
SMD	−35.16	−34.87
SML	−39.79	−38.23
T20	−39.68	−38.23
T60	−35.94	−37.64
T80	−33.28	−37.64

RL and SF [38,46]; OGP, DDGP, DM, SMD and SML [39,51,54]; T20, T60, and T80 [50].

**Table 8:** The values of Gibbs standard free energy of surfactant adsorption at the solid-water interface ($\Delta G_{ads}^{o}$) calculated at *T* = 293 K from: (a) Langmuir equation, (b) linear form of Langmuir equation, (c) Equation (2) and (d) Equation (33).

Surfactant	$\Delta G_{ads}^{o}$, [kJ/mol]					
	Eq.	PTFE-W	PE-W	PMMA-W	nylon 6-W	Quartz-W
RL	a	−42.84	−42.74	−43.41	−42.65	−43.02
	b	−43.91	−43.69	−42.27	−46.93	−48.99
	c	−58.50	−58.66	−34.38	−27.59	−21.12
	d	−52.14	−45.03	−38.99	−31.05	−15.05
SF	a	−51.94	−51.40	−49.72	−42.65	−50.48
	b	−54.02	−53.46	−54.18	−54.8	−55.74
	c	−56.75	−57.34	−33.76	−27.00	−20.77
	d	−61.17	−60.44	−38.30	−26.62	−14.89
OGP	a	−28.60	−28.79	−29.99	−30.20	−30.34
	b	−29.42	−36.25	−32.21	−32.40	−33.44
	c	−32.31	−32.25	−19.44	−15.81	−12.14
	d	−29.90	−32.35	−20.33	−18.51	−6.75
DDGP	a	−42.61	−42.60	−44.03	−45.25	−44.81
	b	−43.37	−44.30	−46.15	−46.64	−47.36
	c	−45.77	−45.91	−29.60	−24.55	−19.11
	d	−41.65	−40.86	−34.32	−30.51	−18.72
DM	a	−39.17	−39.48	−39.94	−41.52	−40.63
	b	−42.33	−42.73	−44.76	−45.60	−45.81
	c	−45.36	−45.43	−29.28	−24.30	−18.90
	d	−41.77	−41.80	−33.45	−29.86	−21.48
SMD	a	−34.54	−34.87	−35.47	−36.08	−31.59
	b	−37.39	−37.80	−38.69	−38.95	−33.99
	c	−47.47	−47.57	−27.81	−22.31	−17.08
	d	−36.80	−37.00	−27.47	−23.94	−15.15
SML	a	−38.91	−38.97	−39.39	−40.76	−39.86
	b	−41.20	−42.19	−43.50	−43.92	−44.41
	c	−47.41	−47.53	−27.99	−22.51	−17.28
	d	−40.59	−39.41	−32.18	−28.53	−20.26
T20	a	−37.33	−37.33	−36.55	−37.67	−37.45
	b	−40.83	−40.83	−40.03	−41.32	−41.23
	c	−55.79	−57.68	−32.56	−25.22	−19.47
	d	−39.71	−39.70	−29.99	−25.12	−12.36
T60	a	−37.49	−36.47	−36.83	−36.15	−36.34
	b	−39.77	−39.41	−39.81	−39.37	−39.55
	c	−74.66	−78.28	−43.30	−32.70	−24.74
	d	−39.01	−39.02	−30.50	−26.89	−18.49
T80	a	−34.32	−34.62	−33.95	−32.79	−33.23
	b	−35.57	−35.36	−37.03	−37.50	−36.47
	c	−77.00	−80.66	−45.69	−34.87	−26.50
	d	−37.83	−37.85	−30.95	−27.93	−21.75

RL and SF [40,41,48]; OGP, DDGP, DM, SMD and SML [40,41,55]; T20, T60 and T80 [49].

**Table 9:** The values of Gibbs free energy of micellization ($\Delta G_{mic}^{o}$) calculated from Equation (34) and the Gibbs free energy of interactions ($\bar{\Delta G_{\mathrm{int}}}$) calculated from Equation (18) at *T* = 293 K.

Surfactant	$\Delta G_{mic}^{o}$ [kJ/mol]	$\bar{\Delta G_{\mathrm{int}}}$ [kJ/mol]
RL	−33.81	−32.35
SF	−37.91	−32.47
OGP	−18.70	−20.34
DDGP	−31.77	−34.56
DM	−30.77	−34.74
SMD	−24.88	−26.83
SML	−29.35	−28.10
T20	−26.67	−28.17
T60	−27.32	−28.14
T80	−27.97	−32.32

**Table 10:** The values of surfactant molar volumes calculated from the density and theoretically based on the bonds length and the angle between them as well as the average distance between molecules at *T* = 293 K.

Surfactant	Molar Weight [M]	Theoretical Partial Minimal Molar Volume [dm^3^/mol]	Theoretical Partial Maximal Molar Volume [dm^3^/mol]	Partial Molar Volume from Density (before CMC) [dm^3^/mol]	Partial Molar Volume from Density (after CMC) [dm^3^/mol]
RL	504.00	407.15	469.43	406.27	471.06
SF	1036.34	1047.40	1296.58	1038.31	1279.51
OGP	292.37	250.87	278.92	244.18	253.12
DDGP	348.47	303.82	343.66	300.42	315.87
DM	510.62	428.79	468.62	416.73	431.08
SMD	496.55	414.43	451.08	377.44	385.92
SML	524.60	440.91	483.45	424.45	436.04
T20	1228.00	1139.85	1216.33	978.52	1085.72
T60	1311.70	1223.36	1313.45	1162.19	1175.05
T80	1310.00	1221.49	1275.65	1021.88	1129.31
